# Factors associated with normal childbirth in Bangladesh: insights from BDHS 2022

**DOI:** 10.1186/s12889-026-27399-w

**Published:** 2026-04-24

**Authors:** Sharmin Akther, Mariam Tonni, Shajid Ahmed, Md. Al-Mamun

**Affiliations:** 1 Department of Statistics and Data Science, Jahangirnagar University, Dhaka, Bangladesh; 2 Faculty of Science and Information Technology, Daffodil International University, Dhaka, Bangladesh; 3 Multidisciplinary Action Research (MARS) Lab, Department of Computer Science and Engineering, Daffodil International University, Dhaka, Bangladesh

**Keywords:** Normal vaginal delivery, Socio-demographic factors, Antenatal care, Socioeconomic status, Multivariable Logistic regression analysis

## Abstract

**Aim:**

The mode of childbirth has severe consequences on maternal and neonatal outcomes. Although the number of cesarean births has grown all over the world, the reasons that make normal vaginal delivery (NVD) a good choice in Bangladesh are under-researched. This research paper analyzes socio-demographic, obstetric, and medical factors that influence NVD to develop specific interventions that can lead to safer and more physiological deliveries.

**Method:**

Data were extracted using 5,006(weighted) ever-married women aged between 15–49 years with live birth within 2 years before the Bangladesh Demographic and Health Survey (BDHS) 2022. Bivariate screening was done using Rao-Scott adjusted chi-square tests, and multivariable binary logistic regression was also done to estimate adjusted odds ratios (AORs) with 95% confidence interval (CI). The Hosmer–Lemeshow test and the variance inflation factors were used to measure the model fit and multicollinearity.

**Result:**

Overall, 55.34% of women gave birth normally through vaginal delivery, and 44.66% gave birth through cesarean section. On adjustment, older maternal age was related to decreased odds of normal vaginal birth; women aged 25–34 (AOR = 0.77; 95% CI: 0.62–0.95) and 35–44 years (AOR = 0.53; 95% CI: 0.35–0.79) had lower odds than women aged 15–24 years. Women in Chittagong (AOR = 2.42; 95% CI: 1.63–3.59) and Sylhet (AOR = 2.26; 95% CI: 1.39–3.65) had significantly higher odds of normal vaginal delivery than women in Barisal. Women who were employed were more likely to deliver normally than unemployed women (AOR = 0.77; 95% CI: 0.63–0.94). On the other hand, women in the top wealth quintile (AOR = 0.52, 95% CI: 0.32–0.86) and those with two or more visits to antenatal care were found to have much lower odds of normal vaginal birth. The facility-based delivery was mainly linked to cesarean section.

**Conclusion:**

Normal vaginal birth in Bangladesh is associated with younger age, certain areas, employment, poverty, reduced ANC usage, and home births. Emerging requirement: evidence-based facility practice and equity to reduce the unnecessary cesareans.

## Introduction

Childbirth mode is one of the most important determinants of maternal and newborn health, as it can have short-term and long-term consequences. Worldwide, vaginal or normal birth is seen as the most natural and safe way of giving birth in the case of no complications at hand [1]. Normal delivery has a lower number of maternal complications, accelerates recovery, and enhances neonatal adaptation compared to cesarean section (CS), which is a surgical procedure [2, 3]. Physiologically, delivery of the baby through the vagina favors maternal hormonal equilibrium, maternal-infant attachment, and newborn respiratory adaptation [4]. Therefore, the normal delivery should be made safe and accessible within maternal and child health strategies across the globe.

Despite such benefits, the rates of CS have been increasing steadily, which frequently exceeds the recommended value of 10–15 percent of births given by the World Health Organization (WHO) [5, 6]. Although CS is life-saving in complex pregnancies, its unwarranted practice presents such risks as bleeding, infection, and minimized recovery of mothers, respiratory distress, and distortion of microbiota growth of newborns [7, 8]. In low-resource families, the cost of surgical births only adds to the issue. Thus, it is a social and economic priority, as well as a public health concern, to promote normal births when medically possible [9].

There has been a significant improvement in the maternal and child health indicators in Bangladesh, such as maternal mortality, antenatal care use, and institutional delivery coverage [10, 11]. But the increased use of cesarean section is now a paradox. According to national surveys, the CS rates have sharply increased, especially in the facilities that are privately run, way beyond global recommendations [12]. This not only poses a threat to maternal health by subjecting them to unnecessary treatment but also results in financial strain on the families. It is also important to promote normal delivery in those settings to protect the health of the mother and maintain the viability of healthcare systems [13].

Normal delivery promotion directly supports Sustainable Development Goal (SDG) 3, which aims to reduce maternal mortality to below 70 per 100,000 live births and neonatal mortality to 12 per 1,000 live births by 2030 [14]. Promoting vaginal delivery contributes to these targets by reducing surgical complications that drive maternal deaths, minimizing neonatal respiratory distress from non-labor cesareans, and decreasing financial barriers to safe delivery. However, while existing research in Bangladesh has documented rising CS rates and identified associated socioeconomic disparities, geographic variations [15], and facility-based determinants of cesarean use [16], the analytical focus has largely remained on cesarean section as the primary outcome. A growing body of evidence suggests, however, that the factors enabling normal childbirth are not simply the inverse of cesarean risk factors. Understanding what facilitates normal delivery requires examining a distinct set of conditions, including supportive intrapartum care, provider adherence to clinical guidelines, and health system readiness for vaginal birth, that may operate independently of factors that drive cesarean use. This distinction has important implications for maternal health programming, as strategies to reduce cesarean rates may not automatically translate into effective promotion of normal childbirth.

A focused examination of factors associated with normal vaginal delivery (NVD) is therefore needed. Earlier studies have identified maternal education as protective against CS [17], prenatal counseling as associated with delivery mode preference [18], and supportive maternity care as linked to vaginal birth outcomes [19]. However, these studies have primarily analyzed such variables as correlates of lower cesarean rates rather than systematically investigating the factors that actively facilitate normal childbirth in Bangladesh. This leaves an important evidence gap: nationally representative studies explicitly modeling the determinants of normal delivery remain limited. The current study addresses this gap by using the Bangladesh Demographic and Health Survey (BDHS) 2022 to identify the socio-demographic, obstetric, and health system factors associated with normal childbirth, providing context-specific evidence to guide interventions that effectively support safe, normal births across Bangladesh.

The main purpose of the research is to find out the socio-demographic, obstetric, and health system variables linked to normal vaginal delivery in Bangladesh using multivariable analysis. This study gives context-specific evidence to direct policymakers, healthcare providers, and maternal health programs in the development of specific interventions by addressing the issue of NVD enablers, which is underrepresented in existing literature. Such interventions will be able to facilitate safe, normal births, decrease unnecessary cesarean deliveries, mitigate socioeconomic and regional inequality, and finally lead to better maternal and neonatal health outcomes in Bangladesh.

## Materials and methods

### Data overview

In this study, the secondary data were utilized based on the most recent Bangladesh Demographic and Health Survey (BDHS) 2022, the ninth installment of the national representative survey in this country. To achieve representativeness in terms of urban and rural settings and across the eight administrative divisions of Bangladesh, the BDHS 2022 used a two-stage stratified sampling technique. The sampling frame consisted of enumeration areas (EAs), which were based on the 2011 Population and Housing Census conducted by the Bangladesh Bureau of Statistics (BBS). The BDHS program carried out all the sampling procedures, and the subsequent data was subjected to the secondary analysis by the authors [20].

### Study participants

The analytic sample was limited to the ever-married women 15–49 years old with a live birth during the two years before the survey. Upon the removal of cases with a missing value in the outcome or on any of the explanatory variables, the final analytic sample comprised 4,965 women (unweighted *n* = 4,965, weighted *n* = 5,006).

### Outcome variable

The primary outcome variable of this study is the mode of delivery, specifically normal (vaginal) delivery. The outcome variable was coded as a binary response: “Yes” for normal delivery and “No” for cesarean section.

### Explanatory variables

**Fig. 1:**
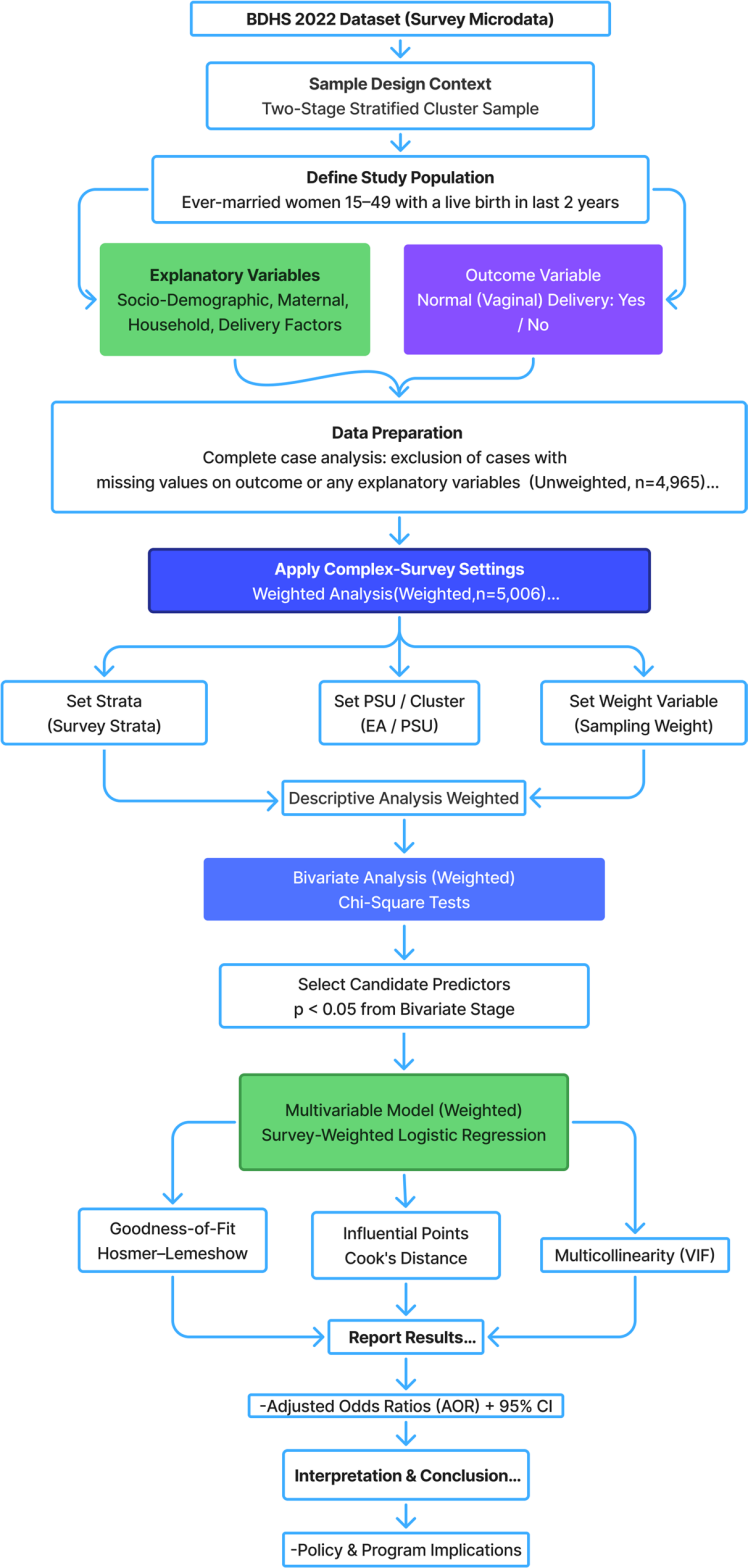
Study design and analytical workflow for identifying factors associated with normal childbirth in Bangladesh

A group of socio-demographic, maternal, and household factors was evaluated as explanatory factors in this investigation. These were chosen based on similar studies [21–25]. These encompassed maternal factors such as maternal age (15–24 years, 25–34 years, 35–44 years, 45 years and above), level of education, working status, parity, antenatal care visits, body mass index, and living children. Household factors involved wealth index, residence place (urban/rural areas), administrative region, sex of household decisionmaker on health care, and household decision on health care. Partner factors involved the husband's level of education. Behavioral and media exposure factors involved newspaper reading frequency as well as TV viewing frequency. These variables of media exposure were considered, as previous research in Bangladesh and other countries of low- and middle-income has demonstrated that a high frequency of television exposure correlates with a higher cesarean section rate, which may be due to creating increased awareness of facility-based delivery services, as well as the influence of television to form a perception of CS as modern or safer [26, 27]. Ultimately, factors related to access to health care involved perception of how far a woman was from a health facility, as well as the delivery place location (home and private facility, public facility, or other health facility). The variable "delivery place" is commonly classified so that 0 represents health facility delivery and 1 represents home delivery. A total of 18 explanatory factors were examined, as listed. The analytical workflow is illustrated in Fig. 1.

### Statistical analysis

All the analyses were conducted considering the convoluted survey composition of the BDHS 2022 to include sampling weights, strata, and primary sampling units (PSUs) to create nationally representative estimates and to modify standard errors to consider clustering and stratification. Descriptive statistics were performed using weights to provide a summary of the characteristics of the participants and the occurrence of normal vaginal delivery. Rao-Scott adjusted chi-square tests were then performed to test the bivariate relationships between the outcome (normal vaginal delivery) and explanatory variables because the survey is a complex design [28]. The multivariate model was chosen using the variables of *p*-value < 0.05 in the bivariate analysis.

Multivariable binary logistic regression was conducted to identify independent factors associated with normal vaginal delivery, reporting adjusted odds ratios (AORs) with 95% confidence intervals. Multicollinearity was evaluated using variance inflation factors (VIF < 10, indicating no issues). Influential observations were assessed with Cook's distance (> 4/n considered influential, where n is the sample size). Model goodness-of-fit was tested using a survey-weighted version of the Hosmer–Lemeshow test, with *p* > 0.05 indicating adequate fit. All data analysis was performed using SPSS version 23.

## Results

**Fig. 2:**
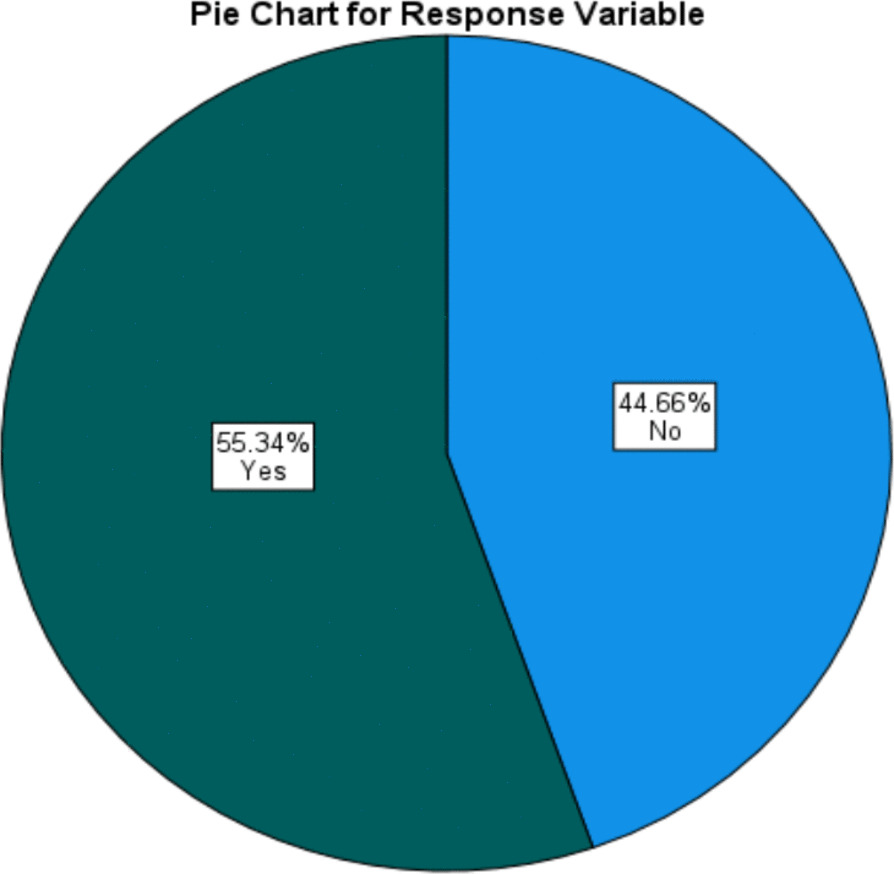
Pie chart based on BDHS 2022 (55.3% normal vaginal delivery vs 44.7% cesarean section)

Among the 5,006 women who were included in the analysis, 55.34% experienced normal delivery, whereas 44.66% delivered by cesarean section, as shown in Fig. 2. Table 1 reflects a summary of the explanatory variables. A total of 5,006 respondents has been identified as having been selected as 18 major variables to be studied. The sample was also characterized by the diversity of the socioeconomic background, with the majority of the participants being located in Dhaka (24.3%) and Chittagong (21.9%). The majority of the women lived in rural locations (73.2%) and were Muslim (92.5%). Almost half (46.9%) of the respondents were 15–24 years and the next group was 25–34 years. Over two-thirds (71.7%) had two or fewer children alive. There was a low rate of employment (77.8% unemployed) and the majority of the households were headed by husbands (88.3%).

**Table 1:** The demographics and background of the study factors

Name	Category	No. of observations	Percentage
Age	15–24	2346	46.9
	25–34	2227	44.5
	35–44	425	8.5
	45 or more	7	0.1
	Total	5006	100
Division	Barisal	313	6.3
	Chittagong	1095	21.9
	Dhaka	1216	24.3
	Khulna	520	10.4
	Mymensingh	444	8.9
	Rajshahi	531	10.6
	Rangpur	560	11.2
	Sylhet	326	6.5
	Total	5006	100
Residence	Urban	1341	26.8
	Rural	3665	73.2
	Total	5006	100
Religion	Muslim	4632	92.5
	Non-Muslim	374	7.5
	Total	5006	100
Education	No education	273	5.5
	Primary	1124	22.5
	Secondary	2716	54.3
	Higher	893	17.8
	Total	5006	100
Husband's education	No education	764	15.3
	Primary	1488	29.7
	Secondary	1778	35.5
	Higher	975	19.5
	Total	5006	100
Sex of household head	Female	584	11.7
	Male	4422	88.3
	Total	5006	100
Health-related decisions	Respondent with others	3241	64.7
	Respondent alone	425	8.5
	Others at home	1340	26.8
	Total	5006	100
Employment	Employed	1114	22.2
	Unemployed	3893	77.8
	Total	5006	100
Parity	Two or less	3590	71.7
	Three or more	1416	28.3
	Total	5006	100
Wealth index	Poorest	1012	20.2
	Poorer	1050	21
	Middle	1029	20.6
	Richer	1002	20
	Richest	913	18.2
	Total	5006	100
ANC visits	No visits	400	8
	One visit	735	14.7
	Two visits	966	19.3
	Three visits	917	18.3
	Four visits	779	15.6
	Five or more	1208	24.1
	Total	5006	100
Distance to health facility	Big problem	2254	45
	Not a big problem	2753	55
	Total	5006	100
Read newspaper	Almost every day	136	2.7
	At least once a week	222	4.4
	Less than once a week	4648	92.9
	Total	5006	100
Watch TV	Almost every day	2290	45.7
	At least once a week	396	7.9
	Less than once a week	2320	46.3
	Total	5006	100
Number of living children	0	64	1.3
	1	1921	38.4
	2	1761	35.2
	3	895	17.9
	4	253	5.1
	5	80	1.6
	6 or more	33	0.7
	Total	5006	100
Mother’s BMI	Normal	1443	28.8
	Obese	2644	52.8
	Overweight	553	11
	Underweight	367	7.3
	Total	5006	100
Delivery place	Health facility	3227	64.5
	Home	1779	35.5
	Total	5006	100

Weighted percentages with BDHS 2022 weights of the survey based on complex survey design (clusters, strata)

The consumption of maternal health services and information sources was still suboptimal. Most women and their husbands had primary or secondary education, which was also related to higher rates of normal vaginal births than those of the more educated women, which is in line with the practice of over-use of cesarean births among more urbanized, educated women in Bangladesh. Only a quarter of women (24.1%) had at any rate of attendance of five or more antenatal care (ANC) visits, and approximately one-fifth had two visits during pregnancy. Over a third of the birth rates were at home (35.5%) and not in a health facility (64.5%). Exposure to mass media was typically low and almost all women read newspapers less than once a week, and almost half watched television less than once a week. Interestingly, more than half or 52.8% of the women were obese by BMI, with the rest being normal, overweight, or underweight. In the bivariate analysis, higher BMI was significantly related to increase in cesarean delivery, which was not statistically significant in the multivariate model after adjustment for other covariates (Table 4).

The adjusted chi-square test by Rao-Scott revealed that mode of delivery was significantly related to many sociodemographic, economic, and healthcare-related factors (*p* < 0.05). As in shown Table 2, it was found that significant factors were geographic division, place of residence (urban versus rural), religion, maternal and husband education, employment, household wealth index, parity, number of children alive, frequency of ANC visits, distance to health facility, autonomy in health-related decision-making, mass media exposure, maternal BMI, and place of delivery. The statistical significance of most of these variables was strong with adjusted chi-square test *p*-values of less than 0.001. The age of the mother also had a statistically significant relationship with the mode of delivery (χ^2^ = 9.719, *p* = 0.035), with women of younger age (15–24 years of age) having a higher prevalence of normal vaginal deliveries whereas women of older age had a higher prevalence of cesarean section. Distinct gradients were found amongst socioeconomic and service-use characteristics. The proportion of normal vaginal delivery was higher among rural women, women with lower levels of education and women who belong to the lower quintile of wealth and women who gave birth at home. On the contrary, urban residence, higher education, higher household wealth, more ANC visits, and facility-based delivery, was found to have a significant positive relationship with cesarean section rates (*p* < 0.001). There were also geographic differences whereby Chittagong and Sylhet had higher rates of normal vaginal labor than Dhaka and Khulna (*p* < 0.001). There was no significant relationship between the mode of delivery and sex of the household head (χ^2^ = 0.069, *p* = 0.819). Since these findings, the total of seventeen variables that proved to be significantly associated during chi-square analysis were then included in the multivariable logistic regression model to determine independent predictors of normal vaginal delivery.

**Table 2:** Relationships between mode of delivery and socio-demographic characteristics

Name	Category	No (C-section)	Yes (Normal)	χ^2^ (*p*-value)
Delivery by Normal Vaginal		2236	2770	
Age	15–24	1062	1284	9.719 (0.035)*
	25–34	1008	1220	
	35–44	165	260	
	45 or more	1	6	
Division	Barisal	118	195	281.968 (< 0.001)***
	Chittagong	338	757	
	Dhaka	648	569	
	Khulna	342	178	
	Mymensingh	174	270	
	Rajshahi	289	242	
	Rangpur	235	325	
	Sylhet	91	235	
Residence	Urban	735	606	75.606 (< 0.001)***
	Rural	1501	2164	
Religion	Muslim	2025	2607	22.496 (0.004)***
	Non-Muslim	211	163	
Education	No education	64	209	346.477 (< 0.001)***
	Primary	328	797	
	Secondary	1241	1475	
	Higher	603	290	
Husband’s education	No education	204	561	384.297 (< 0.001)***
	Primary	508	980	
	Secondary	863	915	
	Higher	660	315	
Sex of household head	Female	258	326	0.069 (0.819)
	Male	1978	2444	
Health-related decisions	Resp. with others	1500	1741	13.622 (0.007)**
	Resp. alone	195	230	
	Others at home	541	799	
Employment	Employed	438	676	16.441 (< 0.001)***
	Unemployed	1798	2094	
Parity	Two or less	1822	1768	189.462 (< 0.001)***
	Three or more	413	1003	
Wealth index	Poorest	231	781	451.859 (< 0.001)***
	Poorer	384	666	
	Middle	466	563	
	Richer	534	468	
	Richest	621	292	
ANC visits	No visits	41	359	510.025 (< 0.001)***
	One visit	179	556	
	Two visits	376	591	
	Three visits	477	441	
	Four visits	417	362	
	Five or more	746	462	
Distance to health facility	Big problem	925	1328	21.358 (< 0.001)***
	Not a big problem	1311	1442	
Frequency of reading newspaper/magazine	Almost every day	93	43	59.428 (< 0.001)***
	At least once/week	136	86	
	Less than weekly	2007	2641	
Frequency of watching TV	Almost every day	1239	1051	175.856 (< 0.001)***
	At least once/week	191	205	
	Less than weekly	806	1514	
Number of children alive	0	15	49	219.627 (< 0.001)***
	1	983	937	
	2	878	882	
	3	292	603	
	4	48	206	
	5	18	62	
	6 or more	2	31	
Mother’s BMI	Normal	613	830	48.905 (< 0.001)***
	Obese	1193	1451	
	Overweight	308	245	
	Underweight	122	245	
Place last child delivered	Health facility	2236	991	2208.722 (< 0.001)***
	Home	0	1779	

*p*-values based on Rao-Scott adjusted chi-square tests (Adjusted F) that adjust the complex two-stage cluster design and sampling weights of BDHS 2022 (*p*-value < 0.05*, *p*-value < 0.01**, *p*-value < 0.001***)

^**^Reference outcome: “Yes” = Normal delivery vs “No” = C-section**

**Fig. 3:**
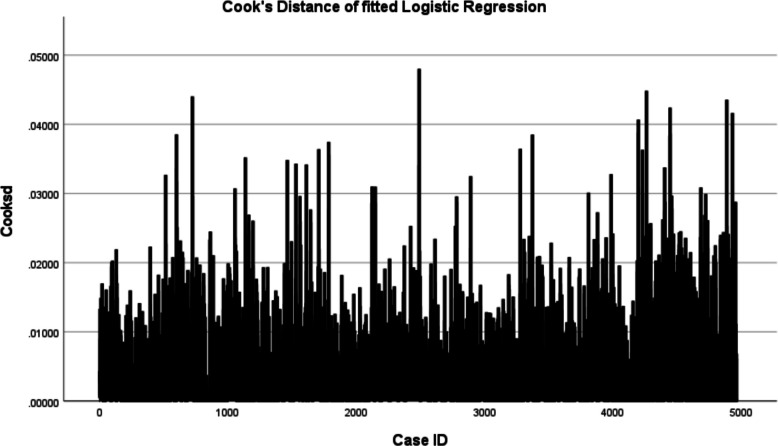
The distance plot created by Cook to show that there are no influential outliers in the multivariable logistic regression model (all values less than 1)

From Table 3, all VIF values ranged between 1.005 and 3.343, well below the accepted threshold of 5, indicating no significant multicollinearity among the covariates. This confirms that the included variables are suitable for the regression analysis and free from multicollinearity issues Fig. 3. Before model fitting, model assumptions were verified. The distance values of Cook were nearly zero, and all of them were significantly less than 1 (Fig. 3), which affirms the absence of influential outliers and provides model robustness to present the logistic regression results of reliable values. These checks provide confidence in the validity of using these predictors in the multivariable logistic regression analysis. To verify the model's assumptions, Hosmer and Lemeshow's goodness-of-fit test was also conducted. For the Hosmer and Lemeshow goodness-of-fit test, the following hypotheses were proposed,

**Table 3:** Covariates VIF values of the logistic regression model

Name	VIF	Df
Age	1.752	3
Division	1.058	7
Residence	1.202	1
Religion	1.032	1
Education	1.675	3
Husband's education	1.662	3
Health-related decisions	1.024	2
Employment	1.052	1
Parity	2.773	1
Wealth index	1.684	4
ANC visits	1.344	5
Distance to health facility	1.03	1
Read newspaper	1.1	2
Watch TV	1.171	2
Number of living children	3.343	6
Mother’s BMI	1.005	3
Delivery place	1.291	1

Variance Inflation Factors (VIF) of multicollinearity test (< 5 = no multicollinearity)

- H_0_: The multivariable logistic regression model fits well with no significant gap between observed and expected frequencies.
- H_1_: The multivariable logistic regression model does not fit well, with no significant gap between observed and expected frequencies.

The analysis yielded a Hosmer–Lemeshow Chi-square value of 2.441 with 8 degrees of freedom and a *p-value* of 0.964, indicating good model fit. The Hosmer–Lemeshow test indicates the logistic regression model fits the data well, as the non-significant *p-value* > 0.05 (0.964) suggests no meaningful discrepancy between observed and expected frequencies in the subgroups.

In the adjusted model (Table 4), the older maternal age (25–34 and 35–44 years), increased household wealth, increased ANC utilization and birth of the child in a health facility had a statistically significant negative relationship with the odds of normal vaginal delivery. Women in the Chittagong and Sylhet divisions were found to be at a much greater risk of normal delivery than their counterparts in Barisal. Other covariates such as maternal and husband education, religion, exposure to media, parity, number of children alive, perceived distance to health facilities and maternal BMI were not significantly related with normal vaginal birth after adjustment. The final model had good fit (Hosmer–Lemeshow *p* = 0.964) and there was no problematic multicollinearity (all VIF < 5). There was evidence of a high association of obesity with mode of delivery in a bivariate analysis, but the BMI categories have not adjusted independently as predictors of normal vaginal delivery. Obese women were borderline more likely to deliver normally than overweight women, and underweight women had no significant differences, suggesting that the obesity cesarean association is not as strong as it appears with socioeconomic and service-use factors.

**Table 4:** Adjusted odds ratios of factors related to normal vaginal delivery based on a multivariable logistic regression analysis

Variable	Adjusted Odds Ratio	95% Confidence	Interval	*p-value*
	**(AOR)**	**Lower**	**Upper**	
Age				
15–24 (ref.)	1.000			
25–34	0.77	0.623	0.952	0.016*
35–44	0.529	0.355	0.789	0.002**
45 +	1.566	0.062	39.245	0.785
Division				
Barisal (ref.)	1.000			
Chittagong	2.421	1.634	3.586	< 0.001***
Dhaka	0.915	0.613	1.366	0.665
Khulna	0.685	0.444	1.057	0.087
Mymensingh	0.772	0.484	1.233	0.279
Rajshahi	0.819	0.53	1.265	0.368
Rangpur	1.038	0.676	1.595	0.863
Sylhet	2.259	1.396	3.655	< 0.001***
Residence				
Urban (ref.)	1.000			
Rural	0.955	0.783	1.165	0.650
Religion				
Muslim (ref.)	1.000			
Non-Muslim	0.99	0.751	1.305	0.942
Education				
No education (ref.)	1.000			
Primary	0.903	0.575	1.419	0.660
Secondary	0.83	0.532	1.293	0.409
Higher	0.77	0.469	1.265	0.302
Husband’s education				
No education (ref.)	1.000			
Primary	0.83	0.621	1.108	0.206
Secondary	0.877	0.657	1.169	0.370
Higher	0.748	0.53	1.056	0.099
Health-related decisions				
Respond with others (ref.)	1.000			
Respond. Alone	0.975	0.723	1.314	0.866
Others at home	1.23	1.021	1.481	0.029
Employment				
Employed (ref.)	1.000			
Unemployed	0.771	0.629	0.945	0.012**
Parity				
Two or fewer (ref.)	1.000			
Three or more	1.387	0.835	2.301	0.206
Wealth index				
Poorest (ref.)	1.000			
Poorer	0.661	0.5	0.874	0.004**
Middle	0.656	0.491	0.874	0.004**
Richer	0.647	0.481	0.87	0.004**
Richest	0.517	0.371	0.721	< 0.001***
ANC visits				
No visits (ref.)	1.000			
One visit	0.837	0.502	1.397	0.496
Two visits	0.579	0.353	0.949	0.03*
Three visits	0.448	0.273	0.737	0.002**
Four visits	0.554	0.337	0.913	0.021*
Five or more	0.524	0.32	0.858	0.01**
Distance to health facility				
Big problem (ref.)	1.000			
Not a big problem	0.935	0.794	1.101	0.419
Read newspaper				
Less than once a week (ref.)	1.000			
At least once a week	0.947	0.521	1.722	0.859
Almost every day	1.026	0.629	1.674	0.918
Watch TV				
Less than once a week (ref.)	1.000			
At least once a week	1.025	0.759	1.385	0.87
Almost every day	0.948	0.792	1.135	0.562
Number of living children				
0 (ref.)	1.000			
1	0.24	0.122	0.473	< 0.001***
2	0.228	0.114	0.453	< 0.001***
3	0.312	0.131	0.744	0.009**
4	0.568	0.22	1.466	0.242
5	0.283	0.088	0.912	0.034
6 or more	1.199	0.176	8.154	0.853
Mother’s BMI				
Normal (ref.)	1.000			
Underweight	1.012	0.84	1.22	0.9
Overweight	0.752	0.565	1.001	0.05*
Obese	1.369	0.98	1.913	0.065
Delivery place				
Home (ref.)	1.000			
Health facility	2.02E + 10	0	0	< 0.001***

Multivariable binary logistic regression using weights of BDHS 2022 survey (*p*-value < 0.05*, *p*-value < 0.01**, *p*-value < 0.001***)

## Discussions

This national representative BDHS 2022 sample shows that slightly more than half of births were delivered through normal vaginal delivery (55.3%), and almost half of births were delivered through a cesarean Sect. (44.7%), a very large share of CS in a low- and middle-income country. This is a huge percentage compared to the recommended population range of 10–15% by the WHO, and it is higher than the present world average of approximately 20–21%. Other recent studies of Bangladesh with BDHS data have also found similar levels, which show a rapid increase in cesarean use over the past 20 years, especially in urban and private hospitals. Adjusted multivariable logistic regression was used to investigate the variables that are related to normal delivery, where 17 variables were identified based on Rao-Scott adjusted chi-square tests.

In contrast to previous descriptive BDHS reports [29–31], our multivariate analysis separates the real independent effect of age. The odds of normal vaginal delivery are significantly less in older women due to the preemptive scheduling of cesarean sections by private facilities in cases estimated as high-risk, which were not previously present in the studies that focused on the population. This trend has existed, although Bangladesh has a predominantly young population of mothers. This is consistent with the results that younger women experience lower complications and operative births. The insignificant result of ≥ 45 years is probably due to the small sample size [32–34]. In biology, the older age of the mother means an increase in hypertensive disorders, gestational diabetes, fetal distress, and labor dystocia, which will reduce the chances of a successful vaginal birth [35, 36]. Medically, old age pregnancies are usually considered to be riskier, and therefore, they are handled more carefully and have a lower decision point to undergo an operation. The non-significant finding in women aged ≥ 45 years may be attributed to a lack of statistical power because of the small sample size.

In contrast to previous BDHS studies [37–39], our 2022 analysis demonstrates that the higher rates of normal vaginal delivery in Chittagong and Sylhet are more likely to be due to weaker penetration of the private hospitals but Barisal and Dhaka are gradually undergoing a change in regional delivery care towards the dominance of the practice by the private-sector, which is evidenced by higher rates in these areas. Urban–rural residence did not show any significant relationship with mode of delivery post-adjustment, probably due to the reduced urban–rural disparity in cesarean delivery rates reported in recent BDHS reports and national studies [39, 40]. Mode of delivery did not have significant independent relationships with maternal and husband education after control, which indicates that their influence could be mediated by socioeconomic status and preference for facilities-based care, which agrees with the earlier evidence in Bangladesh [41–44]. Unemployed women had lower chances of delivering normally than employed women, which indicated that employment could be associated with higher levels of autonomy or availability of maternity care, as other studies had shown before [45, 46].

Although our results suggest a significant odds ratio of the richest-quintile disadvantage with NVD, unlike BDHS studies, which describe more modest odds reductions [45–47], our fully adjusted model estimates a more dramatic decrease in odds, which these previous analyses had underestimated. The given gradient is probably caused by the unequal access to care in the private sector, medical practice trends, and financial or institutional incentives, instead of the impact of medical necessity, and poorer women might be constrained by factors restricting access to facilities that provide care in the home and make them more dependent on home birth [48, 49]. This trend is probably due to unequal access to private healthcare, with a higher rate of cesarean section, which may be caused by providers’ preferences, monetary gain, and maternal desire, but not due to medical necessity [50]. The independent relationship between distance to health facilities was not significant after adjustment, which could be a sign of better geographic accessibility and the availability of the services than in the previous times [51]. ANC repeated visits are counterintuitive predictors of NVD odds due to private facility referral bias, which may be due to repeated contact with facilities, which leads to an increase in referral and institutional delivery, where clinical practice and provider decision-making is biased toward cesarean delivery [52]. Such relationships with the ANC services have been described in previous research [33, 53], especially when ANC services are closely associated with facility-based and private-sector care. These results suggest that there is a need to reinforce evidence-based counseling in the context of antenatal care to make the right delivery choice and minimize unnecessary cesareans [39, 54–56]. Normal vaginal birth was not significantly related to parity at sterilization after adjustment, as reported in prior work [57, 58]. There was no material difference between Muslim and non-Muslim women in normal vaginal birth, as it has already been noted [58]. The media exposure, such as reading newspapers and watching TV, was not significantly related to normal vaginal delivery, consistent with previous studies [58]. Media exposure can be a general indicator of health awareness in Bangladesh, but provider recommendation, facility practices, and socioeconomic access, especially in a private facility, may limit the impact of media exposure on birth outcomes [59, 60]. After adjusting independent variables, none were independently related to parity, religion, media exposure, or BMI, implying that these variables might have an indirect effect on these variables via socioeconomic status and health-service use. As opposed to expectations that facilities enhance results [61, 62], our study reveals systemic discrimination toward normal delivery. Facility-based care is a predictor of near-complete dominance of cesarean sections, which demonstrate that providers opt to prefer surgical intervention over physiological labor, which is absent in guideline-driven systems [63].

Conclusively, normal vaginal birth in Bangladesh was linked to maternal age, region, socioeconomic status, employment, antenatal care use, and place of delivery. The results indicate that socioeconomic status and service uptake attitudes, including facility-based care, are the key factors in determining delivery outcomes. Conversely, education, religion, media exposure, residence, and maternal nutritional status did not have an independent post-adjustment correlation. All in all, the findings suggest the role of systems and institutional practices in the shaping of cesarean use. Evidence-based obstetric care strengthening and advocacy in proper clinical decision-making could help prevent unnecessary cesarean births and provide maternal and neonatal safety.

## Limitations and further studies

This study has used the Bangladesh Demographic and Health Survey (BDHS) 2022, which, though nationally representative, might not be representative of current determinants of normal vaginal birth. Even after controlling for socio-demographic and maternal as well as household variables, there might be residual confounding related to unmeasured variables, including provider habits, cultural tendencies, and facility policies. The effect of facilities on outcomes is not well captured in individual-level data, and an individual cross-sectional design does not support the modeling of provider clustering or allow one to infer causality, but only supports an association. The self-reported data can also cause recall and reporting biases in the accuracy of delivery history. Longitudinal research design should be the main focus of any further research to monitor the changes in service use and clinical practice, with the addition of qualitative research regarding facility settings, incentives to providers, and perceptions of the community. An enhanced approach, such as the multilevel modeling and machine learning, is suggested to reveal intricate hierarchical and non-linear predictors of the delivery modes in Bangladesh.

## Public health implications

The implications of these findings for Bangladesh's maternal health policy are significant. Based on our findings, four accurate and evidence-based interventions are needed. First, DGHS enforces mandatory monthly cesarean audits on Robson-10 in all hospitals with very low risks, but to achieve the prevalence pattern of our NVD, we will start with a pilot in Dhaka. Second, BMNC educates public ANC providers using standardized low-risk NVD-first interventions, focusing on the areas of Chittagong and Sylhet, we find when the odds of NVD are significantly higher. Third, MISIP also adds NVD vouchers to the poorest wealth quintiles (making our rich-poor NVD gap disappear). Fourth, DGHS initiates a real-time Chittagong CS dashboard flagging high-rate facilities to conduct an immediate compliance audit, which also maintains the inherent NVD strength of this division [64, 65].

## Conclusion

This study elucidates the complex interplay of socio-demographic, maternal, household, and delivery factors shaping normal vaginal delivery among women in Bangladesh. Based on the information of BDHS 2022, this research article reveals that the higher rate of normal births is identified among young women, women in the Chittagong and Sylhet divisions, working women, and poor households. On the contrary, higher maternal age, wealth, and antenatal care visits, and institutional birth reduce the probability of vaginal birth denoting that there are robust facility and service variables on mode of delivery. These findings invoke policy intervention to reduce the socioeconomic disparity of quality delivery care, improve evidence-based obstetric care, and regulate unnecessary cesarean deliveries. Scaling up working models of publicly funded maternity, compulsory audit of CS, and standardized counseling on antenatal care can increase informed maternal choice. Such systemic interventions would lessen the morbidity in the surgery, prevent financial burden, and improve the maternal health outcomes in Bangladesh.

## Data Availability

The dataset used in this study was obtained from publicly available sources provided by the *Bangladesh Demographic and Health Survey* ([https://dhsprogram.com/data/available-datasets.cfm] (https:/dhsprogram.com/data/available-datasets.cfm)). All data used for analysis are open access and can be freely downloaded. The processed dataset used in this study is available from the corresponding author upon reasonable request.

## Declarations

This manuscript is an original work and has not been published previously, nor is it under consideration for publication elsewhere, in whole or in part, in any language.
